# Self-Organizing Neural Grove for Malware Detection in IoT Edge Devices †

**DOI:** 10.3390/s26113399

**Published:** 2026-05-27

**Authors:** Hirotaka Inoue, Tsukasa Komura, Ibuki Hashimoto

**Affiliations:** 1 Department of Electrical Engineering and Computer Science, National Institute of Technology (KOSEN), Kure College, 2-2-11 Agaminami, Kure-shi 737-8506, Hiroshima, Japan; 2 Advanced Course, National Institute of Technology (KOSEN), Kure College, 2-2-11 Agaminami, Kure-shi 737-8506, Hiroshima, Japan

**Keywords:** ensemble learning, improving generalization capability, self-organization, edge computing, cybersecurity

## Abstract

Deep learning models, particularly convolutional neural networks (CNNs), have demonstrated exceptional classification performance across various practical applications. However, their training time scales significantly with network depth, rendering them suboptimal for resource-constrained environments. As a practical alternative, multiple classifier systems (MCSs) based on self-generating neural trees provide faster training and lower computational overhead. In this study, we propose the Self-Organizing Neural Grove (SONG), an ensemble learning model featuring a novel pruning technique designed to optimize classification efficiency. We evaluate SONG’s performance on a Raspberry Pi 3, a standard edge computing platform. Through comparative experiments against an unpruned MCS, a C4.5-based MCS, and the k-nearest neighbors (k-NN) algorithm, we demonstrate that SONG achieves superior classification accuracy while substantially reducing both computation time and memory footprint. These advantages are consistent across benchmark datasets and real-world cybersecurity tasks, underscoring the high suitability of SONG for edge computing applications.

## 1. Introduction

Effective classifiers are essential for extracting latent information from large-scale datasets and accurately categorizing previously unseen instances [1]. Recently, multiple classifier systems (MCSs)—such as neural network ensembles [2], bagging [3], and boosting [4]—have been extensively deployed in practical applications to enhance classification accuracy [5] within these MCSs rely on conventional models, including neural networks (e.g., backpropagation and radial basis function networks) [6] and decision trees (e.g., CART and C4.5) [7,8].

Neural networks offer significant advantages, including adaptability, flexibility, and the capacity to approximate complex nonlinear mappings. However, their deployment requires the manual configuration of network architectures and hyperparameters, a process that typically demands domain expertise. Furthermore, training neural networks is computationally intensive, and selecting an optimal network structure for a specific task remains notoriously challenging. These inherent limitations restrict their practicality as base classifiers in MCSs for real-world applications.

Recent literature has extensively explored machine learning and deep learning frameworks for IoT malware detection. For instance, deep autoencoders, recurrent neural networks (RNNs), and convolutional neural networks (CNNs) have been widely deployed to analyze complex network traffic patterns, such as those provided in the N-BaIoT dataset [9,10]. While these deep learning-based approaches achieve exceptional detection rates, their significant computational overhead, intensive training times, and heavy memory footprints present critical bottlenecks for continuous, real-time deployment directly on resource-constrained IoT edge nodes [11].

To mitigate these hardware limitations, recent research has increasingly shifted toward lightweight edge-based classification approaches, commonly categorized under TinyML frameworks, model quantization, and compact tree-based ensembles [12,13]. Although specialized models—such as restricted Random Forests, quantized decision trees, or micro-machine learning (MicroML) architectures—significantly reduce processing requirements, they often introduce a rigid trade-off between classification accuracy and resource efficiency. The proposed SONG framework effectively addresses this research gap by implementing a unique two-stage pruning strategy. Unlike conventional lightweight models that uniformly compress structures at the expense of performance, SONG adaptively optimizes its tree hierarchy based on class-label distributions, thereby maintaining an exceptionally minimal physical memory footprint while fully preserving the robust generalization and classification capabilities of an ensemble system.

Self-generating neural networks (SGNNs) [14], an extension of Kohonen’s self-organizing maps (SOMs) [15], address some of these issues by offering a simpler network design and faster training. SGNNs utilize competitive learning and are implemented as self-generating neural trees (SGNTs) [16]. These characteristics make SGNNs well-suited as base classifiers in MCSs. To enhance the classification performance of SGNNs, we previously proposed ensemble self-generating neural networks (ESGNNs) [17], which aggregate multiple SGNNs in an ensemble framework. Although ESGNNs improve classification accuracy, they also increase computational cost, both in terms of training time and memory usage, proportionally to the number of SGNNs employed.

To resolve these challenges, we introduced a novel pruning method and proposed the Self-Organizing Neural Grove (SONG) [18]. This manuscript is a substantially extended version of our preliminary work presented at the 2025 IEEE 13th International Conference on Intelligent Data Acquisition and Advanced Computing Systems (IDAACS 2025) [19]. While the conference paper focused primarily on the foundational architecture of SONG, this study significantly expands the experimental validation. Specifically, the novel contributions of this extended work are summarized as follows:

- Comprehensive Hardware Evaluation: We conducted an extensive evaluation of SONG on the Raspberry Pi 3, a widely used edge computing platform, to demonstrate its practical applicability in real-world scenarios. This evaluation includes detailed analyses of classification accuracy, inference time, and memory usage, providing a holistic view of SONG’s performance in resource-constrained environments.
- Application to Real-World Cybersecurity: We extended the experimental scope beyond standard benchmark datasets by applying SONG to IoT malware detection using the N-BaIoT dataset, demonstrating its capability to classify malicious traffic generated by Mirai and BASHLITE (Gafgyt) botnets effectively.
- Expanded Comparative Analysis: We strengthened the evaluation by introducing a modern, lightweight comparative baseline (a resource-constrained Random Forest) alongside the k-NN algorithm. This provides a deeper analysis of the trade-offs between classification accuracy, physical memory footprint, and inference time.
- Refinement of Memory Optimization Metrics: We introduced a concrete conversion mechanism mapping structural unit counts to physical memory consumption in bytes, offering practical, transparent guidelines for practitioners deploying models on edge devices.

## 2. Constructing Self-Organizing Neural Grove (SONG)

This section details the pruning strategies employed in constructing the Self-Organizing Neural Grove (SONG). First, we describe the online pruning mechanism applied during the learning phase of the Self-Generating Neural Tree (SGNT). Subsequently, we explain the structural optimization method. Finally, a two-dimensional classification example is presented to illustrate the efficacy of the proposed pruning approach.

### 2.1. On-Line Pruning of the Self-Generating Neural Tree

The Self-Generating Neural Network (SGNN), implemented as a Self-Generating Neural Tree (SGNT), is an extension of Kohonen’s Self-Organizing Map (SOM) [15]. The SGNT is constructed directly from training data without manual intervention, functioning as a hierarchical clustering algorithm where the terminal leaves represent individual training instances.

We define the following notations:

- Input data vector: $e_{i}\in R^{m}$;
- Node (including root and leaves): $n_{j}$;
- Weight vector of node $n_{j}$: $w_{j}\in R^{m}$;
- Number of leaves under $n_{j}$: $c_{j}$;
- Distance measure: $d(e_{i},w_{j})$;
- Winning leaf for input $e_{i}$: $n_{\mathrm{win}}$.

**Figure 1:**
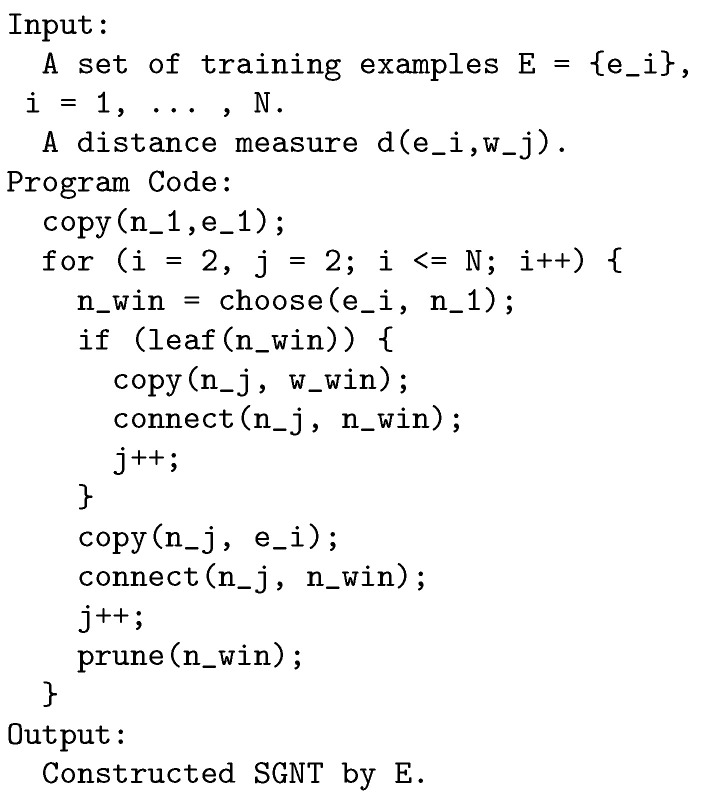
SGNT algorithm.

The SGNT generation process is summarized in Figure 1, with subprocedures described in Table 1. The competitive learning strategy determines the winner node recursively from the root to the leaves. When a node $n_{j}$ contains the winner $n_{\mathrm{win}}$ in its subtree, the weight vector is updated as

(1) $$w_{jk}\leftarrow w_{jk}+\frac{1}{c_{j}}\left(e_{ik}-w_{jk}\right),\;1\leq k\leq m.$$

After all input vectors are assigned as leaf nodes, class labels are assigned to each leaf, and the internal node weights become the average of their descendants. The SGNT’s topology reflects the structure of the input feature space.

To better manage memory and improve generalization performance, we replace the original distance-based threshold for node creation [14] with a novel pruning criterion based on class labels. In the prune($n_{\mathrm{win}}$) subprocedure, if all child leaves of a given node share the identical class, these leaves are pruned, and the corresponding class label is directly assigned to the parent node.

### 2.2. Optimization of the SONG

While SGNTs facilitate rapid learning, their classification accuracy is frequently inferior to that of instance-based methods, such as the *k*-nearest neighbors (*k*-NN) algorithm [20]. This discrepancy arises from the lack of guaranteed proximity to the true nearest leaf for unseen data. To mitigate this limitation, SONG is constructed by aggregating the outputs of multiple SGNTs via majority voting, thereby enhancing overall classification accuracy.

**Figure 2:**
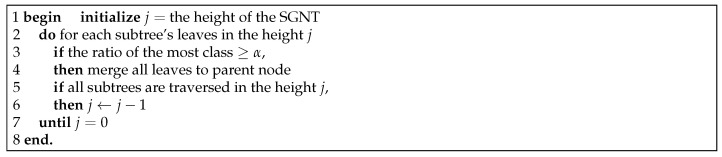
The merge phase.

**Figure 3:**
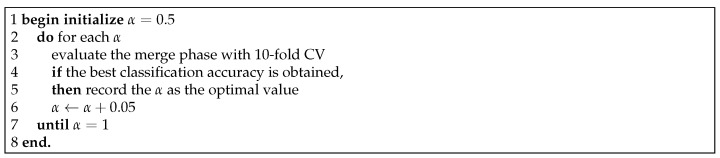
The evaluation phase.

However, increasing the number of constituent SGNTs incurs higher computational and memory overheads, which can impede scalability, particularly on resource-constrained edge devices. To address this trade-off, we propose a two-phase pruning strategy:

- Merge Phase: Dense leaves are pruned if the majority class ratio among sibling leaves under a common parent exceeds a predefined threshold α (see Figure 2).
- Evaluation Phase: The optimal threshold α is determined dynamically via 10-fold cross-validation [21] to strike a balance between classification accuracy and model complexity (see Figure 3).

## 3. Experimental Results

### 3.1. Classification Problems

In this subsection, we evaluate the computational overhead (i.e., memory footprint and inference time) and classification accuracy of the proposed SONG model. The evaluation was conducted using bagging across ten benchmark datasets sourced from the UCI Machine Learning Repository [22] (summarized in Table 2).

To assess the effectiveness of the pruning strategy, we employed 10-fold cross-validation for all datasets. In both the SONG and the *k*-nearest neighbor (*k*-NN) classifier, we utilized a modified Euclidean distance measure defined as follows:

(2) $$\begin{array}{cc} d(x,y) & =\sqrt{\sum_{i=1}^{m}a_{i}\cdot {\left(x_{i}-y_{i}\right)}^{2}}, \end{array}$$

(3) $$\begin{array}{cc} a_{i} & =\frac{1}{\max_{j}-\min_{j}},\;\left(1\leq j\leq N\right), \end{array}$$

where $a_{i}$ serves as a normalization coefficient based on the range of each attribute.

The number of base classifiers (SGNTs) in the SONG ensemble was set to K=25. Each experimental configuration was repeated for 100 trials, with the training data shuffled in a different order for each run to ensure statistical robustness.

All experiments were executed on a Raspberry Pi 3 Model B+, functioning as a representative edge computing environment.

#### 3.1.1. Performance Evaluation of SONG

Table 3 presents the average memory footprint and classification accuracy of the proposed SONG framework across 100 independent trials. Memory consumption is quantified by the total unit count, defined as the sum of root, internal, and leaf nodes within the constituent SGNTs. Assuming a standard 32-bit architecture, each node unit consumes approximately 108 bytes to store pointers, weights, and structural metadata. Consequently, a model comprising 100 units requires a memory footprint of only about 10.8 KB. By establishing this direct conversion, the unit count serves as an accurate and practical proxy for physical memory consumption in edge deployments. The memory consumption of each unit varies depending on the number of features. For instance, in the balance-scale dataset with 4 features, each unit requires approximately 44 bytes, while in the letter dataset with 16 features, each unit requires approximately 140 bytes. The reported memory requirements reflect the average across all SGNTs in the ensemble.

The application of the pruning mechanism significantly reduced the average memory footprint by 65.0% to 96.6% across the evaluated datasets. Concurrently, classification accuracy exhibited improvements ranging from 0.1% to 2.9%. These results demonstrate that the structural optimization process effectively enhances both memory efficiency and predictive performance, validating SONG’s suitability for resource-constrained edge computing scenarios.

To further validate the effectiveness of SONG, we compared its performance with a conventional Multiple Classifier System (MCS) constructed using decision trees (C4.5). For both SONG and the C4.5-based MCS, the number of base classifiers was fixed at K=25, and bagging was employed for ensemble construction. Table 4 presents the classification accuracies obtained from these two models, averaged over 100 trials.

The SONG outperformed the C4.5-based MCS on 6 out of the 10 benchmark datasets. Notably, while the C4.5-based ensemble degraded performance on the iris dataset, SONG consistently improved classification accuracy across all evaluated problems. These findings suggest that SONG provides superior generalization capabilities, particularly on noisy datasets, while maintaining structural scalability.

To ensure the statistical reliability of the observed performance improvements, we conducted a paired *t*-test comparing the classification accuracies of SONG across the 100 independent trials. The statistical analysis confirmed that the accuracy improvements achieved by SONG are statistically significant (*p* < 0.05) for all of the 10 datasets, demonstrating that the performance gains are robust and not due to random variance.

#### 3.1.2. Comparison with *k*-NN

To highlight the practical advantages of SONG, we benchmarked its performance against the classical *k*-nearest neighbors (*k*-NN) classifier. For SONG, we selected the highest classification accuracy from 100 bagging trials. For *k*-NN, we performed 10-fold cross-validation and selected the best accuracy among k∈{1,3,5,7,9,11,13,15,25}.

All experiments were executed on the same edge device, a Raspberry Pi 3 Model B+, with source code compiled using gcc with the -O2 optimization flag. Table 5 shows the classification accuracy, memory usage, and computation time for both SONG and *k*-NN.

While there exist model compression techniques for *k*-NN [23], they typically require considerable computational overhead during training. In our experiments, we used an exhaustive implementation of *k*-NN. Because *k*-NN retains all training samples, its memory requirement scales directly with the size of the training set.

All reported results for *k*-NN reflect averages over 10-fold cross-validation, following the same evaluation protocol as for SONG.

We further analyze the performance of SONG and *k*-NN across three key criteria: classification accuracy, memory requirement, and computation time.

As indicated in Table 5, SONG achieved higher classification accuracy than *k*-NN on 8 out of the 10 datasets, yielding an average improvement of 1.1%. This underscores SONG’s ability to provide more reliable generalization across diverse data distributions.

Furthermore, while SONG incurs initial computational costs for constructing and evaluating *K* SGNT models, its average inference time remains substantially shorter than that of *k*-NN for most datasets. For larger datasets, such as letter, SONG demonstrated approximately 2.33 times faster computation. Because *k*-NN retains all training samples, its memory footprint scales linearly with the dataset size. Conversely, SONG’s efficient node structure maintains a consistently lower memory requirement, an attribute highly advantageous for IoT and edge deployments.

The hyperparameter tuning required for *k*-NN (e.g., selecting optimal *k*) through repeated 10-fold cross-validation can be computationally expensive, especially on large datasets. In contrast, SONG, based on compact and efficient SGNTs, offers a more practical alternative. Furthermore, due to the independence of base classifiers, SONG supports parallel computation, which further enhances its scalability and suitability for deployment on edge devices.

In conclusion, SONG demonstrates favorable trade-offs among classification performance, memory efficiency, and computational cost. These characteristics make it a promising approach for large-scale data mining tasks, especially in resource-constrained environments, as compared to conventional methods such as *k*-NN.

### 3.2. Cybersecurity Applications for IoT Endpoint Devices

To validate the practical applicability of the proposed approach as a cybersecurity countermeasure for IoT endpoint devices, we conducted experiments utilizing the N-BaIoT dataset [24]. The evaluation focused on network communication logs from a specific IoT device model within the dataset, specifically analyzing traffic generated during attacks by two prominent malware variants: Mirai and Gafgyt. Alongside benign (normal) traffic, the dataset comprises representative malicious traffic patterns, including ACK Scan, SYN Scan, UDP Flood, UDP Plain Flood, TCP Flood, Junk Flood, and Combo attacks. These attack vectors, which primarily execute reconnaissance or denial-of-service (DoS) campaigns, represent prevalent threats in real-world IoT deployments. Data subsets corresponding to various attack types were selected for each malware variant, assigned to distinct classes, and integrated with the benign traffic records for classification. The analytical methodology remained consistent with the procedures outlined in the previous experiments.

#### 3.2.1. Data Preprocessing

This subsection aims to clarify the data preprocessing procedure applied to the N-BaIoT dataset used in this paper on IoT botnet attack detection. In particular, it describes the process and rationale for reducing the original 115 feature columns to 35 selected features. To ensure transparency of the processing pipeline and to enable reviewers and replication researchers to reconstruct the dataset under identical conditions, the selection criteria, exclusion criteria, and processing steps are documented in detail.

#### 3.2.2. Data Overview

N-BaIoT (Network-Based detection of IoT botnet attacks) is a publicly available dataset constructed by a research group at Ben-Gurion University of the Negev, Israel [9]. Nine types of real IoT devices—including cameras, smart outlets, and doorbells—were infected with two botnet malware families, Mirai and BASHLITE, and network traffic was recorded both before and after infection. Table 6 summarizes the dataset, which consists of 11 CSV files corresponding to different device and attack combinations. Each file contains network traffic data with 115 features extracted from packet captures, including various statistical and protocol-specific attributes relevant for intrusion detection tasks.

Each filename follows the convention “device_number_botnet_type_attack_type.csv”. Specifically, 1_benign contains normal traffic, 1_gafgyt_ contains BASHLITE (GAFGYT) attack traffic, and 1_mirai_* contains Mirai attack traffic. All 11 files were merged to construct the training and evaluation dataset.

#### 3.2.3. Feature Selection

The original dataset contains 115 features, which include various network traffic attributes such as packet sizes, inter-arrival times, and protocol-specific metrics. To optimize the performance of the classification model and reduce computational overhead, we applied a feature selection process based on the following statistical criteria:

- Mean: The most fundamental statistic representing the center of a distribution. Captures shifts in traffic behavior during attacks.
- Standard Deviation: Represents the spread of a distribution. Traffic uniformity differs markedly between normal and attack conditions.

Through this process, we reduced the feature set from 115 to 35 features, which were deemed most relevant for the classification of IoT malware attacks. This feature selection step was crucial for improving the efficiency of the classification model while maintaining high accuracy, particularly in the context of edge computing environments where computational resources are limited.

#### 3.2.4. Preliminary Classification Experiment

Prior to the primary evaluation, a preliminary classification experiment was conducted utilizing the *k*-nearest neighbors (*k*-NN) algorithm to establish a performance baseline. The sample size was incrementally increased from 100 to 1000 instances (in increments of 100). In our preliminary experiments using small sample sizes (100 to 1000 instances), the k-NN algorithm achieved a classification accuracy approaching 100%. However, this exceptionally high accuracy was strictly limited to simplified binary classification tasks (i.e., benign vs. a single aggregated attack class) under highly controlled and balanced conditions. In contrast, the primary evaluation presented in the next section involves a much more complex multi-class classification scenario using the full dataset, which includes highly imbalanced real-world traffic patterns across multiple specific attack vectors (e.g., ACK Scan, SYN Scan, UDP Flood). This shift from a simplified binary problem to a realistic, multi-class imbalanced problem naturally explains the significant drop in k-NN’s performance to approximately 65%, underscoring the necessity of more robust models like SONG for practical IoT environments.

#### 3.2.5. Experimental Results

In this paper, we focused on evaluating the performance of SONG in comparison to *k*-NN and Random Forest [25], which are commonly used classifiers in cybersecurity applications. Table 7 and Table 8 detail the performance metrics for the classification of the Gafgyt and Mirai malware attacks, respectively. While the baseline k-NN algorithm yielded an average classification accuracy of approximately 65%, the proposed SONG framework significantly outperformed it, achieving 92.86% for Gafgyt and 89.13% for Mirai. Furthermore, SONG maintained a substantially lower memory footprint and superior computational efficiency, demonstrating its robustness in handling malicious traffic classification. Notably, Random Forest classifier achieved near-perfect accuracy (99.98% for Gafgyt and 99.95% for Mirai) but at the cost of increased memory usage and longer computation times compared to SONG. In Random Forest, the depth of each decision tree was set to 5, and the number of trees was set to 25. These results underscore the practical advantages of SONG for real-time IoT malware detection, particularly in resource-constrained edge environments where both accuracy and efficiency are critical.

## 4. Discussion

This study demonstrates that the Self-Organizing Neural Grove (SONG) effectively bridges the gap between high classification accuracy and strict computational limits in edge computing environments. While conventional models like unpruned SGNTs or traditional C4.5 ensembles struggle with memory bloat, SONG’s label-based pruning strategy maintains a compact memory footprint without sacrificing the generalization capabilities of an ensemble approach.

When compared to state-of-the-art lightweight models, SONG offers a highly competitive trade-off. For instance, while the Random Forest classifier achieved near-perfect accuracy on the N-BaIoT dataset (99.98% for Gafgyt), it required significantly more memory and longer computation times than SONG. In resource-constrained IoT endpoints where continuous, real-time monitoring is required, SONG’s balance of high accuracy (over 89%) and minimal memory usage (approx. 42 units) makes it a highly practical alternative.

However, SONG is not without limitations. As observed in Table 5, for exceptionally small datasets such as the glass dataset (214 instances), the inference time of k-NN (0.02 s) was faster than that of SONG (0.25 s). Because k-NN’s computational cost scales linearly with the number of training samples, it holds a speed advantage when the search space is trivial. Conversely, in realistic scenarios involving massive datasets like letter or continuous IoT traffic, k-NN’s overhead becomes prohibitive, whereas SONG maintains superior scalability.

Future work will focus on expanding SONG’s applicability beyond IoT malware detection. Given its parallelizable architecture and efficient memory scaling, we plan to investigate its deployment in other real-time, resource-limited domains, such as autonomous driving sensors and smart infrastructure anomaly detection.

## 5. Conclusions

In this study, we investigated the performance of a multiple classifier system (MCS) based on Self-Generating Neural Trees (SGNTs), termed the Self-Organizing Neural Grove (SONG). We evaluated its computational overhead and classification accuracy on a resource-constrained edge computing platform. To enhance operational efficiency, we introduced a two-stage pruning strategy comprising online learning optimization and offline structural optimization.

Experimental evaluations, including 10-fold cross-validation on benchmark datasets, demonstrated that employing pruned SGNTs as base classifiers significantly reduces memory consumption while simultaneously improving classification accuracy compared to traditional methods like k-NN. Furthermore, experiments targeting IoT malware detection using the N-BaIoT dataset verified SONG’s practical utility in real-world cybersecurity scenarios. The framework successfully reconciled the demand for high classification accuracy with the strict computational limits of IoT endpoint devices.

These findings establish SONG as an effective, scalable, and highly practical MCS framework for large-scale data classification tasks in edge AI environments. As a direction for future work, we plan to explore parallel and distributed implementations of SONG to further extend its scalability. Additionally, we aim to evaluate the framework against a broader range of zero-day attack vectors and investigate its applicability to other domains demanding strict real-time processing, such as autonomous driving and smart infrastructure systems.

## Figures and Tables

**Table 1:** Sub procedures of the SGNT algorithm.

Sub Procedure	Specification
$copy(n_{j},e_{i}/w_{win})$	Create $n_{j}$, copy $e_{i}$/$w_{win}$ as $w_{j}$ in $n_{j}$.
$choose(e_{i},n_{1})$	Decide $n_{win}$ for $e_{i}$.
$leaf(n_{win})$	Check $n_{win}$ whether $n_{win}$ is a leaf or not.
$connect(n_{j},n_{win})$	Connect $n_{j}$ as a child leaf of $n_{win}$.
$prune(n_{win})$	Prune leaves if the leaves have the same class.

**Table 2:** The brief summary of the datasets. *N* is the number of instances, *m* is the number of attributes.

Dataset	*N*	*m*	Classes
balance-scale	625	4	3
breast-cancer-w	699	9	2
glass	214	9	6
ionosphere	351	34	2
iris	150	4	3
letter	20,000	16	26
liver-disorders	345	6	2
new-thyroid	215	5	3
pima-diabetes	768	8	2
wine	178	13	3

**Table 3:** The average memory requirement and classification accuracy of 100 trials for the bagged SGNT in the SONG. The standard deviation is given inside the bracket on classification accuracy ($\times 10^{-3}$).

	Memory Requirement			Classification Accuracy		
Dataset	Pruned	Unpruned	Ratio	Pruned	Unpruned	Ratio
balance-scale	112.38	861.12	13.1	0.87 (5.69)	0.847 (7.82)	+2.3
breast-cancer-w	28.59	897.43	3.19	0.972 (2.24)	0.968 (2.6)	+0.4
glass	104.11	297.77	35.0	0.721 (11.7)	0.716 (12.8)	+0.5
ionosphere	54.3	472.14	11.5	0.893 (7.48)	0.868 (7.79)	+2.5
iris	15.11	208.68	7.24	0.964 (4.55)	0.961 (4.74)	+0.3
letter	6225.08	27,028.56	23.0	0.956 (0.8)	0.955 (0.72)	+0.1
liver-disorders	154.58	471.73	32.77	0.625 (14.12)	0.608 (16.97)	+1.7
new-thyroid	49.55	298.23	16.61	0.952 (6.32)	0.949 (6.76)	+0.3
pima-diabetes	205.9	1045.18	19.7	0.75 (7.27)	0.73 (8.71)	+2.0
wine	14.31	239.04	5.99	0.965 (4.95)	0.96 (5.57)	+0.5
Average	696.39	3181.99	16.8	0.867	0.856	+1.1

**Table 4:** The pruned MCS and the MCS based on C4.5 with bagging.

	MCS Based on SGNT			MCS Based on C4.5		
Dataset	SGNT	MCS	Ratio	C4.5	MCS	Ratio
balance-scale	0.782	**0.87**	+8.8	0.795	0.827	+3.2
breast-cancer-w	0.957	**0.972**	+1.5	0.946	0.963	+1.7
glass	0.641	0.721	+8	0.664	**0.757**	+9.3
ionosphere	0.853	0.893	+4	0.897	**0.92**	+2.3
iris	0.949	**0.964**	+1.5	0.953	0.947	−0.6
letter	0.879	**0.956**	+7.7	0.880	0.938	+5.8
liver-disorders	0.58	0.625	+4.5	0.635	**0.736**	+10.1
new-thyroid	0.935	**0.952**	+1.7	0.93	0.94	+1
pima-diabetes	0.7	0.75	+5.0	0.749	**0.767**	+1.8
wine	0.95	**0.965**	+1.5	0.927	0.949	+2.2
Average	0.823	0.869	+4.4	0.837	**0.874**	+3

The bold values indicate the best performance between the pruned MCS based on SGNT and the MCS based on C4.5 for each dataset.

**Table 5:** The classification accuracy, the memory requirement, and the computation time of 100 trials for the best pruned SONG and *k*-NN.

	Classification Acc.		Memory Requirement		Computation Time (s)	
Dataset	SONG	k-NN	SONG	k-NN	SONG	k-NN
balance-scale	0.883	**0.899**	**114.46**	562.5	**0.55**	0.85
breast-cancer-w	**0.976**	0.973	**26.67**	629.1	0.79	**0.51**
glass	**0.756**	0.706	**115.97**	192.6	0.25	**0.02**
ionosphere	**0.909**	0.857	**53.87**	315.9	1.53	**0.21**
iris	**0.973**	0.96	**13.49**	135	0.09	**0.02**
letter	0.958	**0.961**	**6214.2**	18,000	**167.27**	389.76
liver-disorders	**0.664**	0.647	**199.37**	310.5	0.35	**0.2**
new-thyroid	**0.968**	0.967	**53.57**	193.5	0.17	**0.01**
pima-diabetes	**0.766**	0.753	**188.46**	691.2	**1.26**	1.28
wine	**0.983**	0.977	**10.86**	160.2	0.2	**0.05**
Average	**0.884**	0.873	**699.09**	2119.1	**17.25**	39.29

The bold values indicate the best performance between the pruned SONG and *k*-NN for each dataset.

**Table 6:** Summary of the N-BaIoT dataset.

Filename	Botnet Type	Attack Type
1_benign.csv	None	Benign
1_gafgyt_scan.csv	GAFGYT	Scan
1_gafgyt_udp.csv	GAFGYT	UDP Flood
1_gafgyt_tcp.csv	GAFGYT	TCP Flood
1_gafgyt_junk.csv	GAFGYT	Junk Flood
1_gafgyt_combo.csv	GAFGYT	Combo
1_mirai_ack.csv	Mirai	ACK Scan
1_mirai_syn.csv	Mirai	SYN Scan
1_mirai_scan.csv	Mirai	Scan
1_mirai_udp.csv	Mirai	UDP Flood
1_mirai_udpplain.csv	Mirai	UDP Plain Flood

**Table 7:** Comparative performance of the SONG, *k*-NN, and Random Forest for the classification of Gafgyt attack types.

Gafgyt	SONG	*k*NN	Random Forest
acc. (%)	92.86	65.25	**99.98**
memory req. (units)	**42.8**	90	133
comp. time (s)	0.306	**0.096**	0.552

The bold values indicate the best performance among the three classifiers for each malware type.

**Table 8:** Comparative performance of the SONG, *k*-NN, and Random Forest for the classification of Mirai attack types.

Mirai	SONG	*k*NN	Random Forest
acc. (%)	89.13	64.38	**99.95**
memory req. (units)	**42.24**	90	100
comp. time (s)	0.272	**0.099**	0.544

The bold values indicate the best performance among the three classifiers for each malware type.

## Data Availability

Data is applicable in the UCI Machine Learning Repository: https://archive.ics.uci.edu/ml/index.php (accessed on 24 May 2026). The N-BaIoT dataset is available at https://www.kaggle.com/datasets/mkashifn/nbaiot-dataset?select=2.benign.csv (accessed on 24 May 2026).
